# Species Composition, Structure, and Regeneration Status of Woody Plants and Anthropogenic Disturbances in Zijje Maryam Church Forest, Ethiopia

**DOI:** 10.1155/2022/8607003

**Published:** 2022-12-02

**Authors:** Amare B. Mekonnen, Wubetie A. Wassie, Habtemaryam Ayalew, Berhane G. Gebreegziabher

**Affiliations:** ^1^Bahir Dar University, College of Science, Department of Biology, Bahir Dar, Ethiopia; ^2^Woldia University, Faculty of Natural and Computational Sciences, Department of Biology, Weldiya, Ethiopia

## Abstract

Our current study was conducted in Zijje Maryam Church Forest, Ethiopia, to explore woody species composition, structure, regeneration status, and anthropogenic disturbances inside the sacred groves. The aforementioned information for adequate conservation and management of the church forest is not well documented. Fifteen main quadrats each having an area of 625 m^2^ (25 m × 25 m) were used for vegetation and disturbance data collection. Determination of the sampled quadrats was based on the principle that minimum quadrats give the smallest possible area in which all species occurring in the church forest are present. All woody species with a diameter at breast height (DBH) ≥ 2.5 cm within the quadrat were identified, counted, and their height and DBH data were recorded. The criterion to start at DBH ≥ 2.5 cm was to exclude seedlings having DBH < 2.5 cm and height ≤0.6 m. Sapling and seedling data were collected using 45 saplings and 45 seedling quadrat that measured 4 m^2^ and 1 m^2^, respectively. Vegetation data analysis and ANOVA were used for statistical comparison. A total of 48 woody plant species belonging to 46 genera and 36 families were identified. *Fabaceae* was the dominant family containing 5 species followed by *Rosaceae* with 3 species. Total basal area of the church forest was 83.03 m^2^ ha^−1^. The density of seedlings, saplings, and matured woody species stem ha^−1^ were 15555, 3833, and 865, respectively. Talking these densities, the regeneration status of the forest was good. The Shannon diversity and evenness of woody plant species in the forest was high, 3.29 and 0.85, respectively. *Juniperus procera* 27.67 (9.22%) and *Olea europaea* were species with the highest IVI. Nearly, 22% of areas of the forest get disturbed and higher anthropogenic disturbances occurred near the edge of the forest. Gathering, clearing, and grazing are the major human disturbances that stakeholders need to tackle for conservation. Zijje Maryam Church Forest has heterogeneous species composition with varied seedlings and saplings. Therefore, local conservation policies recommended not only protect large forests, but also the small and valuable forests service to the needs of local people.

## 1. Introduction

Biodiversity refers to the variety and variability among living organisms and the ecological complexes in which they occur. The term encompasses different ecosystems, species, and their relative richness and abundance [1]. According to [2], biodiversity is a variety of life at genetic, species, and ecosystem levels. Biodiversity contributes a lot in one way or another to food production and agriculture. It encompasses the vast range of organisms that live in and around food and agricultural production systems [3].

Species diversity, which is limited to the local or regional level, comprises richness (the number of species) and evenness (the relative number of individuals of each species) and is an important issue in ecology [4]. Biodiversity at genetic, species, and ecosystem levels helps to address the challenges possessed by diverse and changing environmental conditions and socioeconomic circumstances. Many key components of biodiversity for food and agriculture at genetic, species, and ecosystem levels are declining and the threats to biodiversity are increasing. The decline in biodiversity may diminish human wellbeing by decreasing the services that ecosystems can provide for people [5].

Biodiversity in forests includes essential and valuable resources because plant species and their genetic reserves are highly significant in providing for the needs of mankind and other organisms, and the lack of biodiversity is a dangerous threat to those needs [6]. Different factors in an ecosystem contribute to plant biodiversity [7]. But, the distribution of plant species is more influenced by topography in mountainous regions [8]. Biodiversity in the tropics is threatened by intense anthropogenic pleasure. Deforestation, habitat degradation, habitat fragmentation, exploitation, invasive species pollution, global climate change, and synergies among them have had a major impact on biodiversity [9].

According to [10], forests are the single most important repositories of terrestrial biodiversity. They provide a wide range of products and services worldwide. They preserve water and sustain the existence of other organisms including humans [3]. They provide aerated habitat for animals, prevent soil erosion, and mitigate climate change [11]. Forests are climate change mitigates because they soak up carbon dioxide and other greenhouse gases that would otherwise be free in the atmosphere and contribute to ongoing changes in climate patterns. Three hundred million people worldwide live in forests and 1.6 billion depend on them for their livelihoods [12]. Forests protect our watersheds, provide places for recreation, and supply oxygen [13]. Forests also provide a wide array of services such as timber production, climate stabilization, regulation of water quantity and quality, and cultural benefits [14].

Ethiopia is rich in biodiversity with a wide range of ecological conditions ranging from arid lowlands in the east to rainforest in the west and high altitude Afro-alpine vegetation in the central highlands [15]. Forests and trees in Ethiopia have played an important role in providing a variety of economic beneficial goods such as fuel wood, construction materials, food, and fodder [16]. In addition to their environmental functions, these renewable natural resources also contribute significantly to the social, psychological, and cultural expressions of Ethiopian people's livelihood [17]. Deforestation in the highlands of Ethiopia remains process dating back many hundreds of years resulting in patches of forest (only areas covered by vegetation) mainly around religious centers in inaccessible and protected areas [18].

When we come to the status of sacred forests, natural sacred sites exist in many countries all over the world except Antarctica. A review by [19] on sacred groves potential for biodiversity management indicated that, 33 countries are identified as having sacred groves—three each from North and South America, five from Europe, twelve from Africa, eight from Asia, and two from Oceania. But, the numbers of sacred groves in Ethiopia are not stated in the review [19]. Locally, the Ethiopian Orthodox Tewahido Church has a long history of forest conservation practices due to religious thought and local people's willingness to protect and conserve them [20]. Ethiopian sacred forests are an important repository place of biodiversity, some of which are endemic and endangered, and thus are an essential mechanism of biodiversity conservation [19]. Within Ethiopia where the majority of the landscape is deforested, the sacred forests make up a significant component of forest habitat [20]. The increasing threats to these forest ecosystems lead to study the current status in species composition, density, diversity, structure, regeneration status, and anthropogenic disturbances of Zijje Maryam Church Forest, Ethiopia. This study is novel in providing baseline information on Ethiopian sacred groves potential for biodiversity management.

## 2. Materials and Methods

### 2.1. Description of the Study Area

**Figure 1:**
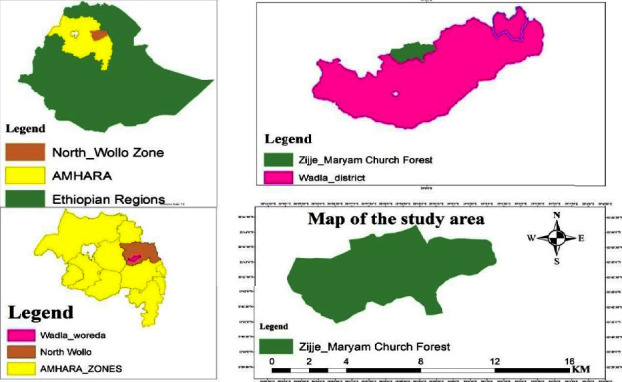
Location map of the study area.

The study was conducted in Zijje Maryam Church Forest in Wadla District, North Wollo Zone, Amhara Regional State, Ethiopia (Figure 1). The study site is located 558 kilometers far away from Bahir Dar, the capital city of Amhara Region, and 617 kilometers far away from Addis Ababa, the capital city of Ethiopia, through the streets from Addis Ababa, Dessie, Woldia, and Gashena. Wadla District is located on 11°29′29.82″N to 11°41′25.53″N latitude and 39°02′19.19″E to 39°19′53.74″E longitude. The altitude ranges from 700 m to 3200 m above sea level [21].

### 2.2. Climate

**Figure 2:**
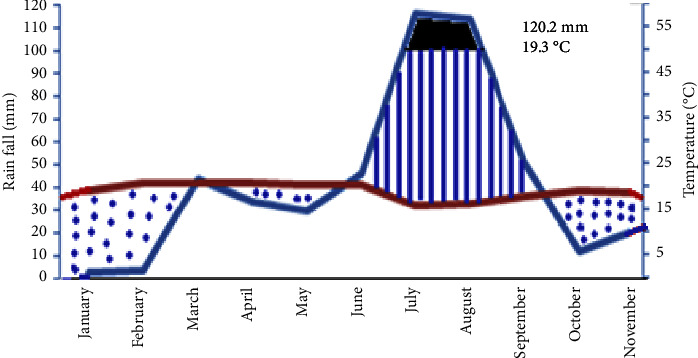
Average rainfall and temperature data of 10 years (2009–2018) of Wadla District modified using the 2020 National Meteorological Data of Kombolcha Station.

The annual maximum temperature for Wadla ranges from 20.18°C–28.46°C. The minimum temperature of the district ranges from 11.51°C–13.82°C. The average annual rainfall ranges from 2.59 mm–264.39 mm as indicated from the diagram (Figure 2).

### 2.3. Sampling Design

Systematic sampling methods [22] were used to set up the line transects and quadrats to collect both vegetation and disturbance data. Line transects were laid down in 10 meters apart from the church in three directions surrounding the church in the midpoint; that means, three modified transects were established from the inner edge of the church forest at 0°, 120°, and 240° in each of the 3 compass directions following [23]. Systematic sampling was used to lay a total of 15 quadrats on the above three transects to collect vegetation data from the study site. Five quadrats were laid along each transect line. The first line transect was laid systematically from the church to 0° north of the mountain. Fifteen quadrats having an equal size of 25 m × 25 m (625 m^2^) were laid down by considering the church as a midpoint to collect vegetation and disturbance data following [24]. The number of sampling quadrats was determined based on the size of the definite area, the church forest cover an area which was 0.2 hectare in average, because differences in quadrat sizes potentially led to differences in species richness as the probability of finding additional species increases with increasing quadrat size. The vegetation data of each woody plant and human disturbances in the quadrats were counted and measured following [25, 26], respectively.

The distances between the main quadrats were 40 m along each of the line transects. To collect seedling data, three subquadrats with the size of 1 m^2^ (1 m × 1 m) within each main quadrat were used. The subquadrats were arranged systematically, two at the corners and one in the middle of each quadrat following [27]. To collect the data, on the other hand, three subquadrats were used in each main quadrat. These subquadrats measured 4 m^2^ (2 m × 2 m) following [27]. Two of the subquadrats were laid at the corner and one was laid in the middle of the main quadrat.

### 2.4. Vegetation Data Collection

Vegetation data were collected and recorded in from each of the fifteen laid quadrats. The season of data collection was accompanied from October to December 2021. The abundance and density of all woody plants with DBH ≥ 2.5 cm in each quadrat were identified, counted, and registered; their DBH and height were measured. DBH was measured at 1.3 m from the ground using a rolling meter. The criterion to start measuring woody plants at ≥2.5 cm was to exclude the seedlings having DBH < 2.5 cm and height ≤0.6 m [28]. Plant identification was done in the field by recording the vernacular (local) names of identified woody plant species, and the numbers of individual plant species were recorded. However, plant species that were difficult to identify in the field were collected, pressed, dried, and mounted and then brought to Bahir Dar University for identification by experts of BDU by referring [16] and following flora of Ethiopia and Eritrea [29–31]. The voucher specimens were identified and then kept at Bahir Dar University Biology Department.

To determine the diameter at breast height (DBH), the circumference of each woody species at breast height, i.e., 1.3 m from the ground were measured by using a rolling meter and then changed into DBH (circumference = 2*πr*, and DBH = circumference/*π*). The circumferences of each woody plant greater than or equal to 7.85 cm (DBH ≥ 2.5 cm) were measured using a measuring tape following [15]. The height of trees and shrubs were measured using marked bamboo sticks. For certain difficulties encountered to measure the height, visual estimations were also used.

To determine the regeneration status of woody plants of the forest, the type and density of seedlings and saplings of woody plants were determined. Vegetation data of seedlings and saplings were collected from subquadrats. The numbers of all seedlings that measured ≤1 m height were recorded and individual plants with the height ≥1 m but DBH less than 2.5 cm were considered as saplings and all of them were counted and recorded [32–57].

### 2.5. Disturbance Data Collection

The occurrences of the two major types of disturbances (human and natural disturbances) were collected in the main quadrats in areas they cover in m^2^. The areas of human disturbance like logging (clearings), gathering areas (Mahiber), grave and grave houses, human trails, buildings, plantation (native and exotic species), and grazed part of the plants for grazing were measured and calculated for each quadrat. The areas of natural disturbances (e.g., rocky and wind fallen tree) covered in the quadrats were measured and collected. The amount of natural disturbances was insignificant which affect only 1.6% of the area of the forest as compared to human disturbance which was 20.53%. Thus, the total disturbances were taken as human disturbance due to insignificance of natural disturbance and the same scale of measurement, i.e., the area of disturbance cover (m^2^) was used. This was the reason behind for pooling together different disturbances status into one. The percentage area covered by each category of disturbances in each quadrat was calculated [26]. The percentage area covered by each type of disturbance was calculated by the sum of the areas of each disturbance in a quadrat divided by the area of the quadrat. Then, it was multiplied by 100 to change into percentages.

(1) $$\begin{array}{c} \%\;Area\;covered\;by\;di\;sturbance=\frac{Area\;covered\;by\;di\;sturbance\;in\;a\;quadr\;at}{Area\;of\;quadr\;at}*100\text{.} \end{array}$$

Using this data, the status of disturbance of the church forest was determined and the edge effect of the forest was evaluated. The positions of quadrats (inner, middle, or edge) were disturbed for human disturbances. The quadrats were arranged from the innermost (quadrat-5) to the outer side of the forest or the edge of the forest (quadrat-1) in three different directions following [26]. To determine the distance between the edge of the forest and first quadrat, it was designed as 30 m far from each other from the inner to the edge of the forest within the center of each of the three transects (between 10 m from the outer edge and 10 m from the internal wall around the church).

### 2.6. Data Analysis

#### 2.6.1. Species Diversity and Evenness

The species diversity and evenness of the study area was quantified using Shannon and Weiner species diversity and evenness index. This method is one of the most widely used approaches in measuring plant species diversity and evenness. The Shannon index is an information statistic index, which means it assumes all species are represented in a sample and that they are randomly sampled [33]. It was computed as follows:

(2) $$\begin{array}{c} H^{\prime }=-\overset{S}{\underset{i=1}{\sum }}pi\;\ln\;pi\text{,} \end{array}$$

where *H*′ is Shannon–Wiener index, *Pi* is the proportion of individual tree species, and *S* is the number of species of that taxonomic group observed.

The woody plant species richness of the study area was expressed as the number of woody plant species collected and enumerated in each quadrat and taken as the sum of all those species, and then their number of families and genus were counted separately.

The Shannon–Wiener evenness was calculated as follows:

(3) $$\begin{array}{c} J=\frac{H^{\prime }}{H^{\prime }\;\max}=\frac{H^{\prime }}{\ln\left(S\right)}\text{,} \end{array}$$

where *J* = species evenness (equitability), *H*′ = Shannon–Wiener diversity index, and *H*′ max =ln(*S*) where *S* is the number of species. *S* = the number of species of that taxonomic group observed.

#### 2.6.2. Data Analysis for Structural Parameters

The plant species were recorded for each quadrat according to their habits as trees, shrubs, and woody climbers (lianas) and explained in percentages. The abundance for each woody species was determined as the total area coverage of each species in each quadrat. The vegetation structure was described by calculating the diameter at breast height (DBH), cover abundance, tree density, height, frequency, and important value index. The following structural parameters were used to calculate the value of vegetation structure.

*(1) Basal Area*. It is the area outline of a plant at the breast height surface, i.e., at 1.3 m height. It is expressed in square m/hectare [34].

(4) $$\begin{array}{c} Basal\;area\left(m^{2}\right)=\frac{{\pi d}^{2}}{4}\text{,} \end{array}$$

where *π* = 3.14159265 and *d* = DBH by *m*.

*(2) Diameter at Breast Height (DBH)*. The structural data of DBH was analyzed by classifying them into 10 DBH classes (i.e., DBH class 1 (2.5–12.5 cm), 2 (12.51–22.51 cm), 3 (22.52–32.52 cm), 4 (32.53–42.53 cm), 5 (52.54–62.54 cm), 6 (62.55–72.55 cm), 7 (72.56–82.56 cm), 8 (82.57–92.57 cm), 9 (92.58–102.58 cm), and 10 (>102.58 cm)) following [27].

*(3) Density*. It is a count of the number of individuals of a species within the quadrat [35]. Afterwards, the sums of individuals per species were calculated and analyzed in terms of species density per hectare [34].

(5) $$\begin{array}{c} D\left(Density\right)=\frac{the\;number\;of\;above\;ground\;stems\;of\;species\;counted}{sample\;area\left(ha\right)}\text{,}\\ RD\left(Relative\;Density\right)=\frac{Density\;of\;species\;A}{Total\;de\;nsity\;of\;all\;species}\times 100\text{.} \end{array}$$

*(4) Frequency*. It is defined as the probability or chance of finding a species in a given sample area or quadrat. It depends on quadrat size, plant size, and patterning of the vegetation [35]. It was calculated with the following formula:

(6) $$\begin{array}{c} F\left(Frequency\right)=\frac{the\;number\;of\;quadr\;ats\;where\;which\;that\;species\;ocurs}{The\;total\;number\;of\;quadr\;ats}\times 100\text{.} \end{array}$$

Relative frequency was calculated as follows:

(7) $$\begin{array}{c} Rf\left(Relative\;Frequency\right)=\frac{Frequency\;of\;species\;A}{Total\;frequencyvof\;all\;species}\times 100\text{.} \end{array}$$

*(5) Importance Value Index (IVI)*. IVI was used to compare the overall dominance and ecological significance of species. It combines data for three parameters (relatives; frequency, density, and abundance) or it often reflects the extent of dominance, occurrence, and abundance of a given species in relation to other associated species in an area [35].

(8) $$\begin{array}{c} IVI=RD+Rf+RDO\text{,}\\ Dominance=\frac{total\;basal\;area}{area\;sampled\;by\;ha}\text{.} \end{array}$$

Relative dominance was calculated as follows:

(9) $$\begin{array}{c} RDO\left(RelativeDominance\right)=\frac{do\;minance\;of\;species\;A}{total\;do\;minance\;of\;all\;species}\times 100\text{.} \end{array}$$

*(6) Height*. Individual trees and shrubs having height greater than or equal to 2 m and DBH greater than or equal to 2.5 cm within sampling quadrats were collected, counted, recorded, and analyzed by classifying them into 10 height classes 1 (2.0–4.50 m), 2 (4.51–7.0 m), 3 (7.01–9.50 m), 4 (9.51–12.0 m), 5 (12.01–14.50 m), 6 (14.51–17.0 m), 7 (17.01–19.5 m), 8 (19.51–22.0 m), 9 (22.01–24.50 m), and 10 (>24.50 m) following [27].

### 2.7. Data Analysis for Regeneration Status

Since there are two factors, human disturbance, eight in total and the 15 quadrats distance from the center of the church replicated five times, the researchers want to determine which factor/s mostly impacts the forest and at which distance. Thus, ANOVA was used to decrease type I error instead of *T*-test. Besides error reduction, ANOVA was deliberately used in this study based on the small effect size (where effect size is the size of a statistically significant difference that can be measured as the standardized difference between two means) concept of Cohen that increased autocorrelation coefficient. The regeneration status of sample species of Zijje Maryam Church Forest was analyzed by comparing seedlings with saplings and saplings with matured woody plant species following [36]. The following categories were used to analyze the regeneration status of this church forest. (1) If seedling > sapling > mature tree, it is “good” regeneration status; (2) if seedling > sapling < mature tree, the regeneration status becomes “fair” regeneration; (3) if a species survives only in the sapling stage, but not as seedlings (although saplings may be less than, more than, or equal to mature), the regeneration status of that forest becomes “poor” regeneration; (4) if a species is absent both in sapling and seedling stages, but present as mature, “none”; and (5) if a species has no mature, but only sapling and/or seedling stages, the forest is “new” as noted by [37].

### 2.8. Disturbance Data Analysis

ANOVA using SPSS software was used to test the significant difference between the types of human disturbances and the quadrat distance from the church (center) on the rate of human disturbance across quadrats inside the church forest at *P* < 0.05. Shapiro–Wilk tests based on the correlation of the data and the corresponding normal scores were used and conducted in the SPSS explore procedure (analyze⟶descriptive statistics⟶explore⟶plots⟶normality plots with tests). The scores in the samples were passed the normality test by converting to *Z*-scores using *Z*_Skewness_ and *Z*_kurtosis_ formula as in small samples, values greater or lesser than 1.96 are sufficient to establish normality of the data.

## 3. Results and Discussion

### 3.1. Woody Species Composition

**Figure 3:**
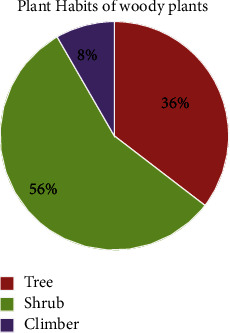
Comparison of the growth forms of woody plant species in Zijje Maryam Church Forest.

A total of 48 woody species belonging to 46 genera and 35 families were recorded from Zijje Maryam Church Forest. Among the collected species, many of the woody species were shrubs, trees, and climbers, respectively (Figure 3). *Fabaceae* was the most dominant family and ranked first; it was the most species rich family contributing 5 (10.42%) species followed by *Rosaceae*. *Rosaceae* ranked second next to *Fabaceae*, which contributed 3 (6.25%) species to the total woody species. Then, *Asteraceae*, *Celastraceae*, *Cupressaceae*, *Lamiaceae*, *Moraceae*, *Sapindaceae*, and *Urticaceae* in which each of the abovementioned families contributed 2 (4.17%) species for the total woody plant species, and these all were the third ranked families next to *Rosaceae*. Each of the rest 26 families contributed 1 (2.08%) species to the total woody species of Zijje Maryam Church Forest (Table 1).

The findings from this study are helpful in providing the new information on the status of sacred forests in Ethiopia that has great contribution to the scientific community. The findings can provide baseline information on Ethiopian sacred grove status in composition, density, diversity, structure, regeneration, and anthropogenic disturbances and their potential for biodiversity management where Ethiopia is missed from the 12 African countries having sacred groves as reviewed by [19].

### 3.2. Species Diversity and Evenness

The results of the present study showed that the species diversity and species evenness of woody plants in the study area were found to be 3.29 and 0.85, respectively. This “comparatively high diversity and evenness reflect the more stable the ecosystem and the better efforts on forest conservation and management in the sacred church forests of the study area. According to [35], the Shannon–Weiner diversity index normally varies between 1.5 and 3.5 and it rarely exceeds 4.5. According to [38], the Shannon diversity index is high when it is above 3.0, medium when it is between 2.0 and 3.0, low when between 1.0 and 2.0, and very low when it is smaller than one. Therefore, the Shannon–Wiener diversity index (*H*′) in this study was 3.29 which was high indicating the forest was rich in diversity. Communities with a large number of species that are evenly distributed are the most diverse, and communities with few species that are dominated by one species are the least diverse as noted by [39]. The Shannon–Wiener diversity index and evenness value of this study was higher than Boda forest diversity (1.79) and evenness (0.1) of northwestern Ethiopia of Montane forest [40]. Similarly, the diversity and evenness of woody species of Zijje Maryam Church Forest was higher than 2.98 and 0.65, respectively, of Tara Gedam and Abebaye forests, northwestern Ethiopia [41]. The possible reason is suggested to be the larger wall fencing of the forest which remained with active conservation efforts of the stakeholders. Therefore, the individuals of woody plant species diversity and evenness in the study area were reflected as more diverse and evenly distributed in the forest.

### 3.3. Vegetation Structure

#### 3.3.1. The Diameter at Breast Height (DBH)

The vegetation structure of trees, shrubs, and lianas/climbers of Zijje Maryam Church Forest were stratified using the DBH class. The patterns of DBH class distribution indicated the general trends of population dynamics and recruitment processes of the species. The distribution of woody plant species in different DBH classes were analyzed by classifying them into 10 DBH classes. Different patterns of species population structure can indicate variation in population dynamics as noted by [42].

**Figure 4:**
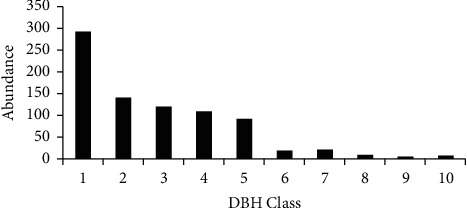
DBH classes of matured woody plant species of Zijje Maryam Church Forest/2020. Key: numbers represent the DBH classes; 1 (2.5–12.5 cm), 2 (12.51–22.51 cm), 3 (22.52–32.52 cm), 4 (32.53–42.53 cm), 5 (52.54–62.54 cm), 6 (62.55–72.55 cm), 7 (72.56–82.56 cm), 8 (82.57–92.57 cm), 9 (92.58–102.58 cm), and 10 (>102.58 cm).

The results revealed that the distribution of woody plant species in the present study showed a high number of individuals in the first DBH class. Plants with DBH class 2 ranked second as far as the number of individual woody plants were concerned. As shown in the graph of the DBH class, the second most abundant individual trees were recorded in DBH class 2. Plants with DBH class 3 were the third most dominant based on the number of individual plants available; whereas small values were registered in the rest DBH classes (Figure 4**)**. The least dominant DBH class of this study was DBH class 9, which was represented by only 4 individual plants. The DBH class map of this study was inverted *J*-shaped (Figure 4). This pattern indicates that the majority of the species had the highest number of individuals with lower DBH and a gradual decrease towards the higher class. This in turn shows that the forest vegetation has good reproduction and recruitment potential. Differently, the DBH class map of Weiramba forest was bell shaped [43], and similar results to the current study were reported by [44, 45]. Such DBH pattern in the forest is a normal population structure and shows the existence of species in healthier conditions as indicated by [46].

### 3.4. Height Class Distribution

**Figure 5:**
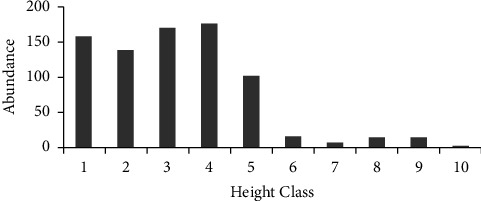
Height class frequency distribution of woody species. Key: numbers represent the height class; (1) (2.0–4.50 m), (2) (4.51–7.0 m), (3) (7.01–9.50 m), (4) (9.51–12.0 m), (5) (12.01–14.50 m), (6) (14.51–17.0 m), (7) (17.01–19.5 m), (8) (19.51–22.0 m), (9) (22.01–24.50 m), and (10) (>24.50 m) of Zijje Maryam Church Forest/2020.

The result indicated that height class 4 contained the highest number of individual woody species of plants that was 21.82% (177 individuals). Therefore the majority of the height classes were distributed under height class 4. Height class 3 was the second highest height class which was represented by 21.09% (171 individuals). Height class 1 was represented by 19.73% (160 individuals). There were a higher number of tree/shrub/climber individuals in the height classes 1, 2, 3, and 4, which accounted for 79.78% (647 individuals) of the total height class. This is due to the distribution pattern, showing that the higher number of individuals at height class 1 occupied better niche, i.e., microenvironment compared with the lower number of individuals at the other height classes. The majority of the woody species (trees, shrubs, and climbers) individuals of this study were distributed in the first DBH class. Different patterns of species population structure can indicate variation in population dynamics as noted by [47]. The rest height classes (height class 5, 6, 7, 8, 9, and 10) were 20.22% (164 individuals) of the total 10 height classes. The last fewer height classes were occupied by fewer numbers of individual species as shown in the graph of height class distribution (Figure 5). The height class distribution map of Weiramba forest was bell-shaped [43]. Similar results to the current study were reported by [47] and [48].

### 3.5. Basal Area

The total basal area of woody plants in Zijje Maryam Church Forest with DBH ≥ 2.5 cm was about 83.03 m^2^ ha^−1^. Basal area provides a measure of the relative importance of the species rather than a simple stem count [24, 49, 50]. The basal area of the study sites compared with some other studies was higher, but still some other studies had a higher basal area. For instance, the BA ha^−1^ of the present study was higher than the studies conducted in Yemrehane Kirstos Church Forest, Weiramba Forest, Magda Forest, and Gedo Forest; that means the basal area per hectare of the present study was higher. The reason for higher basal area in this study would be due to the provision of higher comparative importance of the woody plant species to the study area. Basal area provides the measure of the relative importance of the species rather than simple stem count [24, 28]. However, the BA ha^−1^ of the present study was less than BA ha^−1^ of Menna Angetu forest (Table 2). The total basal area of the current study was higher when compared with the previous studies conducted in Magda Forest, Yemrehane Kirstos Church Forest, Weiramba Forest, and Gedo Forest (Table 2). Species with higher basal area could be considered as the most important species in the study of vegetation. Therefore, species with the largest contribution to the BA can be considered as the most important species in the forest following [50].

In the study conducted in Zijje Maryam Church Forest, basal area analysis across individual species indicated that there was high domination by very few or small woody species. Thus, species with the largest basal area can be considered the most important woody species in the study area. Those individual woody plant species which contributed more to higher basal area in this study include *J. procera* Hochst. (54%), *O. europaea* L. (23.65%), *V. abyssinica* (Hochst. Ex Benth) K. and B. (5.14%), *M. undata* (Thunb.) Blakelock (3.38%), *P. abyssinicum* Del. (2.37%), *H. abyssinica* (Bruce) J. F. Gmelin (1.88%), *G. saxifrage* (Hochst.) Bridson (1.58%), and *M. ovatus* Schweinf. (1.41%), respectively. As far as BA was concerned, *J. procera*, *O. europaea*, and *V. abyssinica* ranked first, second, and third, respectively (Table 3). This suggests that the aforementioned species of Zijje Maryam Church Forest had better growth and potential to retain higher biomass.

### 3.6. Importance Value Index (IVI)

According to [35], the Importance Value Index combines data from three parameters which include RF, RD, and RDO. It is crucial to compare the ecological significance of species [50]. It was also described that species with the greatest importance index are the leading dominant of specified vegetation following [53].

The hierarchies of importance value indices of this study were calculated and ranked. The highest IVI value of the present study was contributed by *J. procera* which was ranked first. As indicated by [50], IVI is used for comparison of ecologically significant species in which high IVI values indicate that the species structure in the community is high. Hence, *J. procera* in this study was high in structure. Its IVI and %IVI were 27.67 and 9.22, respectively. *O. europaea* was the second dominant species of this study. Its IVI and %IVI were 23.09 and 7.70, respectively. As the output of IVI analysis showed that the top 10 woody plant species of this study were *J. procera* Hochst. (9.22%), *O. europaea* L. (7.7%), *M. ovatus* Schweinf. (3.49%), *P. abyssinicum* Del. (3.1%), *V. abyssinica* (Hochst. Ex Benth) K. and B. (2.63%), *E. arborea* L. (2.59%), *G. saxifrage* (Hochst.) Bridson (2.34%), *M. undata* Schweinf. (2.32%), *D. torrid* (J. F. Gmel) Bamps (2.24%), and *U. hypselodenderon* A. Rhi. (2.07%) (Table 4).

### 3.7. Frequency

The present study revealed a high percentage of species in lower frequency classes and a relatively low percentage of the number of species in high frequency classes. Thus, the result verifies the existence of a high degree of floristic heterogeneity in Zijje Maryam Church Forest. The relative frequency revealed that *J. procera* Hochst. was the most frequent species with a frequency of 100, followed by *O. europaea* L., *M. ovatus* Schweinf., *P. abyssinicum* Del., and *M. undata* Schweinf. Similar result was reported from the study conducted in Yemrehane Kirstos Church Forest with the highest density of *J. procera* and *O. europaea*, respectively, but better in the basal area in this study [24].

Frequency is the number of quadrats in which a given species occurred in the study area. The five most frequently observed woody plant species of this study were *J. procera* Hochst. which occurred 100 times out of 15 quadrats which were having 9.74 relative frequencies. The relative frequencies of some most frequent woody plants were *O. europaea* L. 9.09, *M. ovatus* Schweinf. 4.55, *P. abyssinicum* Del. 3.90, and *M. undata* Schweinf. 3.90. The rest 43 frequently occurred species together contributed 68.83% of the total relative frequency of the forest (Table 4). The least occurred species were *R. myricoides* (Hochst.), *A. retinoides*, *R. abyssinica* Lindley., *C. simensis* Fresen., *R. nervosus* (vahl), *R. prinoides* L'Herit., *O. ficus indica* (L) Miller, *F. carica* L., and *D. abyssinica* (A. Rich.) Warb. each having 0.65 relative frequencies and total 5.84 relative frequencies. The possible reason that the authors mentioned could be Table 5 due to less competitiveness and less seedlings and sapling to reach maturity stage of these species compared with the most frequent ones.

### 3.8. Regeneration Status of Zijje Maryam Church Forest

The density of seedlings (15555.56) was greater than saplings (3833.33) and mature trees (865.07) (Tables 6 and 7). Saplings were also greater than mature trees, although they were less than seedlings. As noted by [37], if seedling > sapling > mature tree, it is a “good” regeneration status. Because of the density of seedlings was > the density of saplings > the density of mature trees, the regeneration status of the present study was good.

**Figure 6:**
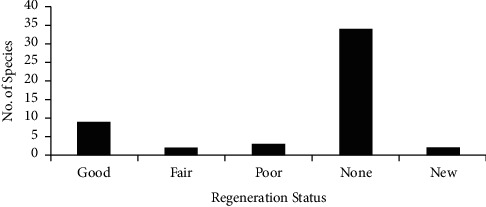
The regeneration status of Zijje Maryam Church Forest.

Based on the results of this study, 9 species (18.75%) of the total woody plant species were represented by both seedling and sapling stages. These species showed “good” regeneration status. Those plants that were represented by both seedlings and saplings were *E. arborea*, *M. ovatus*, *O. lamiifolium*, *R. abyssinica*, *M. kummel*, *D. viscosa*, *F. sycomorus*, *P. abyssinicum*, and *D. torrid* (Tables 5 and 6). There were 3 species (6.25%) that were represented by saplings but not seedlings (Table 6). Those species were *E. schimperi*, *L. aurita*, and *M. salicifolia*. These species showed “poor” regeneration status. The two other species (4.17%) were available in seedlings but not in saplings. These species were *C. aurea* (Ait.) Benth. and *A. abyssinicus* (Table 7). These species showed “fair” regeneration status (Figure 6).

Among the total woody plants, 37 species (77.08%) were not represented by seedlings and 36 species (75%) of the total woody plants were not represented by sapling stages. Other species were such as without seedlings and those without sapling stages. Therefore, these woody plants have lower regeneration status, which may suggest that these species are either under the threat of local extinction or may prefer coppices or sprouts as the strategy of survival. If a species is absent both in sapling and seedling stages, but present as mature, it has no regeneration potential as noted by [37]. Thirty four species (70.83%) of the total woody plant species were represented by neither seedlings nor saplings. These 34 species (70.83%) were not regenerating. There were also new species that were represented by seedling and sapling stages but not in mature stages. *M. africana* and *P. chimperiana* were new species which were not available in the mature stage but in seedlings and saplings. These species showed “new regeneration” status in Zijje Maryam Church Forest (Figure 6).

From the total of 50 woody plant species found in Zijje Maryam Church Forest, 13 species (26%), 14 species (28%), and 48 species (96%) have seedlings, saplings, and matured woody plants, respectively. The following species had the largest contributions to the seedling counts *D. abyssinica*, *P. africana*, *M. ferruginea*, *M. ovatus*, *M. Kummel*, and *C. tomentosa*. The study of the regeneration potential of a forest is one of the major means to assess its conservation status.

The overall regeneration status of the forest was not satisfactory. Most of the woody plants were not regenerated (70.83%). Such conditions might have been occurred due to existing disturbances in the study site like over grazing, firewood collection, and poor biotic potential of tree species which either affect the fruiting or seed germination or successful conversion of seedlings to sapling stage following [27]. The possible reasons for insufficient seedlings and saplings for the above tree species in the forest might be seed predation, grazing and browsing, lack of safe sites for seed recruitment, nature of seeds of certain trees which seek dormancy period, litter accumulation, pathogens, species specificity, and moisture stress or might have other alternative adaptations for propagation and reproduction rather than seed germination [56].

### 3.9. Comparison of Disturbances Based on Their Impact

The relationship between the disturbances and species diversity was determined. This is because a higher species diversity help the forests to have a more stable ecosystem functioning and enhances both productivity and stability [28]. In this study the least disturbed quadrats of the sacred forest were found to have the highest species diversity. The total area coverage by all disturbance type was 2075 m^2^ among 9375 m^2^ area of quadrats studied in the forest. Thus, 2,207 m^2^ha^−1^ of forest land was disturbed. Nearly 22.13% of the areas of the forest get disturbed. This is very low compared to the study of [26] on disturbance coverage of 44 church forests in South Gonder that were 56.2%. This could be because of some of the conservation effort observed in the study area via religious thoughts, religious supports, its inviolability, and legal safeguard (the civil law and guards).

**Figure 7:**
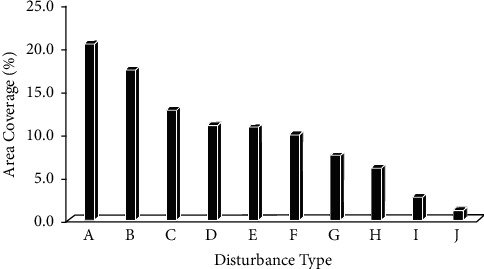
The contribution of the amount of each type of disturbance that affect the church forest; where A = gathering area (Mahiber), B = logging, clearing, C = grazing, D = grave and grave houses, E = buildings, F = trails, G = plantation (exotic species), H = rocky, I = plantation (native species), and J = wind fallen tree.

There was a significant difference (*P* < 0.05) between the types of human disturbances and the quadrat distance from the church (Table 8). The result revealed that the gathering area (Mahiber) was the highest among the ten human disturbances occurred in the church forest which covers nearly 21% of all disturbed area (Figure 7). The impact of gathering area is due to the ceremonial effect throughout the year. This causes lack of safe site for seed recruitment, affects nature of seeds of certain trees which seek dormancy period, affects litter accumulation, affects species specificity, and moisture stress [54]. Probably church forests might have other alternative adaptations for propagation and reproduction rather than seed germination [54]; therefore, impacts the species diversity and evenness. Wind had the least effect followed by logging (clearing), and grazing (Figure 7). The amount of natural disturbances (rocky area and wind fallen trees) was insignificant which affected only 1.6% of the area of the forest compared to human disturbance which was 21%. Thus, the total disturbance can be taken as human disturbance due to the insignificance of natural disturbance. This is analogous to the result of [26].

**Figure 8:**
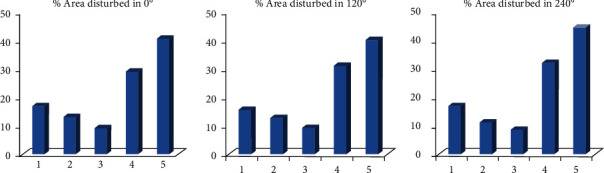
The amount of area of forest disturbed by human disturbance from the inner quadrats (1) to the edge of the church forest (5).

Quadrats near the edges of the church forest were disturbed critically higher than the inner quadrats by human disturbances (Figure 8). Similar result was found by [53] that the edge effect was significantly higher affecting the soil nutrients near the edge of the church forest quadrats. Similarly, [56, 57] also found exotic plantation disturbances across quadrats were significantly different.

The rate of human disturbance critically affected the amount of basal area (m^2^/ha), diversity, evenness, and the number of seedlings found in the quadrats (Figure 8). These disturbances reduced the vegetation density, diversity, evenness, and number of seedlings of woody plants in the forests. Finally, the accepted manuscript has been deposited on a preprint server [58] as per the stated recommendation during the acceptance email from Scientifica to the corresponding author.

## 4. Conclusion

The study confirmed that Zijje Maryam Church Forest was used as a reservoir of high woody species diversity. A total of 48 species belonging to 36 families and 46 genera were recorded. In Zijje Maryam Church Forest, 865.07 density ha^−1^of woody plant species were collected. There were high values of woody plant species in the first frequency class, low values in the middle frequency class, and a simple decline in the last frequency class. Therefore, this declination in those frequency classes indicates that Zijje Maryam Church Forest had heterogeneous species composition. The Shannon–Wiener Diversity Index (*H*′) and evenness of this church forest were high. From the overall distribution of DBH classes in Zijje Maryam Church Forest, the high contribution of woody plant species in the lower DBH and height classes and lower plant contribution in the higher DBH and height classes As the DBH class size increases, the number of individuals gradually decline. Therefore, this provided a regular inverted *J*-shaped DBH and height class distribution. This indicates that there was the dominance of small-sized woody plants in the forest. The data analysis of Zijje Maryam Church Forest revealed that the density values of seedlings (15555.56 density ha^−1^) and saplings (3833.33 density ha^−1^) of the population structure were higher when compared to mature woody plants (865.07 density ha^−1^). This is due to varieties of factors that hinder seedlings and saplings to reach mature stage (death of seedlings and saplings before reaching mature stages). Most woody plants species of Zijje Maryam Church Forest were not regenerating. Therefore, it is recommended, these species need priority for *in situ* conservation measures to be carried out. Critical attention should be given for the forest conservation in the *in situ* since seedlings and saplings were not reaching the mature stage. The policy suggestion for this study would be therefore, indicating the presence of not studied, small but valuable and potential sacred church forests in Ethiopia. Therefore, local conservation policies recommended not only protecting large forests, but also the small and valuable forest services to the needs of local people.

## Figures and Tables

**Table 1:** Top nine families with the number of genera and species in Zijje Maryam Church Forest.

Family	No. of genera	%	No. of species	%
*Fabaceae*	5	10.87	5	10.42
*Rosaceae*	3	6.52	3	6.25
*Celastraceae*	2	4.35	2	4.17
*Asteraceae*	2	4.35	2	4.17
*Cupressaceae*	2	4.35	2	4.17
*Lamiaceae*	2	4.35	2	4.17
*Moraceae*	2	4.35	2	4.17
*Sapindaceae*	2	4.35	2	4.17
*Urticaceae*	2	4.35	2	4.17
Others	24	52.17	26	54.17
Total	46	100.00	48	100.00

**Table 2:** Basal area comparison of the current study with some other studies conducted in Ethiopia where the vegetation data were collected in the same DBH.

Study sites	DBH	Basal area (ha^−1^)	Source
Zijje Maryam Church Forest	—	83.03 m^2^	Current study
Yemrehane Kirstos Church forest	DBH > 2.5 cm	72 m^2^	[24]
Menna Angetu		94.22	[51]
Weiramba Forest	—	32.10 m^2^	[43]
Magda Forest	—	68.52	[16]
Gedo Forest	—	35.45	[52]

**Table 3:** List of woody plant species with high basal area and their percent contribution of BA m^2^ ha^−1^ in Zijje Maryam Church Forest.

Species	BA	BA (m^2^ha^−1^)	%	D (ha^−1^)	%
*Juniperu procera* Hochst.	42.04	44.84	54.00	152.53	17.63
*Olea europaea* L.	18.41	19.64	23.65	118.40	13.69
*Vachelia abyssinica* (Hochst. Ex Benth) K. and B.	4.00	4.27	5.14	36.27	4.19
*Maytenus undata* (Thunb.) Blakelock	2.63	2.80	3.38	20.27	2.34
*Pittosporum abyssinicum* Del.	1.85	1.97	2.37	40.53	4.69
*Hagenia abyssinica* (Bruce) J. F.Gmelin	1.47	1.56	1.88	11.73	1.36
*Galiniera saxifrage (*Hochst.) Bridson	1.23	1.31	1.58	28.80	3.33
*Maytenus ovatus* Schweinf.	1.10	1.17	1.41	45.87	5.30
Others 40 species	5.13	5.47	6.59	410.67	47.47
Total	77.84	83.03	100	865.07	100

Where BA = basal area, BA (m^2^ha^−1^) = basal area m^2^ per hectare, and D (ha^−1^) = density per hectare.

**Table 4:** Importance Value Index (IVI) of the top ten woody plant species of the study area.

Species	RD	RF	RDO	IVI	%IVI	Rank
*Juniperus procera* Hochst.	17.63	9.74	0.29	27.67	9.22	1
*Olea europaea* L.	13.69	9.09	0.31	23.09	7.70	2
*Maytenus ovatus* Schweinf.	5.30	4.55	0.63	10.48	3.49	3
*Pittosporum abyssinicum* Del.	4.69	3.90	0.73	9.31	3.10	4
*Vachelia abyssinica* (Hochst. Ex Benth) K. and B.	4.19	2.60	1.10	7.89	2.63	5
*Erica arborea* L.	4.07	2.60	1.10	7.76	2.59	6
*Galiniera saxifrage* (Hochst.) Bridson	3.33	2.60	1.10	7.02	2.34	7
*Maytenus undata* Schweinf.	2.34	3.90	0.73	6.97	2.32	8
*Dombeyatorrida Dovyalis* (J. F. Gmel) Bamps	2.59	3.25	0.88	6.71	2.24	9
*Urera hypselodenderon* A. Rhi.	2.10	3.25	0.88	6.22	2.07	10
Others 38 species	40.07	54.55	92.25	5.92	62.29	11
Total	100	100	100	300	100	

Where RD = relative density, RF = relative frequency, RDO = relative dominance, IVI = Important Value Index and %IVI = percentage of Importance Value Index.

**Table 5:** Frequency distribution of dominant woody species in Zijje Maryam Church Forest.

Species	No. of quadrats	*F*	RF
*Juniperus procera* Hochst.	15	100	9.74
*Olea europaea* L.	14	93.33	9.09
*Maytenus ovatus* Schweinf.	7	46.67	4.55
*Pittosporum abyssinicum* Del.	6	40	3.90
*Maytenus undata* Schweinf.	6	40	3.90
Others 43 species	106	706.67	68.83
Total	154	1026.67	100.00

Where *F*=frequency and RF = relative frequency.

**Table 6:** Abundance and density per hectare of saplings of Zijje Maryam Church Forest.

Species names	No. of saplings	Sampled area by ha	Density ha^−1^	% density ha^−1^
*Erica arborea* L.	9	0.018	500	13.04
*Maytenus ovatus* Schweinf.	8	0.018	444.44	11.59
*Ocimum lamiifolium* Hochst.ex.Benth.	5	0.018	277.78	7.25
*Euclea schimperi* SchI.	1	0.018	55.56	1.45
*Myrsine afrcana* L.	17	0.018	944.44	24.64
*Rosa abyssinica* Lindley.	2	0.018	111.11	2.90
*Mimusops kummel* A.DC	2	0.018	111.11	2.90
*Laggera aurita* (L.f.) Benth. ex C. B. Clarke	9	0.018	500	13.04
*Dodonaea viscosa* L.	1	0.018	55.56	1.45
*Myrica salicifolia* A. Rich.	1	0.018	55.56	1.45
*Ficus sycomorus* L.	2	0.018	111.11	2.90
*Pentas schimperiana* Schi.	2	0.018	111.11	2.90
*Pittosporum abyssinicum* Del.	3	0.018	166.67	4.35
*Dombeyatorrida Dovyalis* (J. F. Gmel) Bamps	7	0.018	388.89	10.14
Total	69		3833.33	100

**Table 7:** Abundance and density per hectare of seedlings of Zijje Maryam Church Forest.

Species names	No. of seedlings	Sampled area by ha	Density ha^−1^	% density ha^−1^
*Erica arborea*	11	0.0045	2444.44	15.71
*Maytenu sovatus*	8	0.0045	1777.78	11.43
*Ocimum lamiifolium*	3	0.0045	666.67	4.29
*Calpurnia aurea*	2	0.0045	444.44	2.86
*Allophylus abyssinicus*	2	0.0045	444.44	2.86
*Myrsine africana*	27	0.0045	6000	38.57
*Rosa abyssinica*	4	0.0045	888.89	5.71
*Mimusops kummel*	2	0.0045	444.44	2.86
*Dodonaea viscosa*	1	0.0045	222.22	1.43
*Ficus sycomorus*	2	0.0045	444.44	2.86
*Pentas schimperiana*	2	0.0045	444.44	2.86
*Pittosporum abyssinicum*	4	0.0045	888.89	5.71
*Dombeyatorrida dovyalis*	2	0.0045	444.44	2.86
Total	70		15555.56	100

**Table 8:** ANOVA results determining the types of human disturbance and disturbances across quadrats number inside the church forest (*P* < 0.05).

Source of variation	SS	df	MS	*F*	*P* value	*F* crit
Types of human disturbance	6157	7	879.6	14.1	0.00001	2.10
Disturbances across quadrats	9741.9	14	695.8	11.2	0.00001	1.79
Error	6106.2	98	62.3			
Total	22005	119				

## Data Availability

The data used and/or analyzed during the current study are included within the article.
